# Prevalence of COVID-19 Vaccine Side Effects among Early-Vaccinated Healthcare Workers in Eastern Ethiopia

**DOI:** 10.4314/ejhs.v32i3.2

**Published:** 2022-05

**Authors:** Godana Jarso, Wassie Gebi, Meyrema Abdo, Misgana Lemma, Endashaw Abebe, Bekana Lemessa, Biniyam Tefera Deressa

**Affiliations:** 1 Adama Hospital Medical College

**Keywords:** COVID-19, COVID-19 Vaccine, ChAdOx1 nCoV-19 Corona Virus Vaccine, Healthcare workers, Vaccine side effects, Vaccine hesitancy, Adama Hospital Medical College, Ethiopia

## Abstract

**Background:**

The Ministry of Health of Ethiopia launched the COVID-19 vaccination campaign in March 2021, with frontline healthcare workers as first-round recipients and a goal of vaccinating 20% of the population by the end of 2021. The study aims to estimate the prevalence of COVID-19 vaccination side effects among early vaccinated healthcare workers in Adama hospital medical college.

**Methods:**

A cross-sectional study was carried out between March and June 2021, following the vaccination of COVID-19 vaccine among healthcare workers in Adama hospital medical college. The study used a structured self-administered questionnaire and additional telephone surveys on items covering the participants' demographic data, local and systemic manifestations after vaccination.

**Results:**

A total of 540 health care workers and supportive staff were enrolled in this study. The overall any-symptom report after the first dose of ChAdOx1 nCoV- 19 vaccine was 84.3%. The majority (39.6%) of participants had both systemic and local symptoms and 25.7% had only local and 18.9% had only systemic symptoms. Injection site pain was the most prevalent side effect symptom (64.1%), followed by fatigue (35.7%), headache (28.9%), joint pain (26.5%), and muscle pain (21.5%).

**Conclusion:**

Vaccine side effects were common and found to be well-tolerated among the recipients of the first dose of ChAdOx1 nCoV-19 at Adama hospital medical college healthcare workers. The side effects were mainly mild to moderate. More side-effect profiles should be studied and disseminated to detect rare adverse reactions.

## Introduction

The coronavirus disease 2019 (COVID-19) is a communicable respiratory disease caused by a new strain of coronavirus that causes illness in humans(1). COVID-19 was first reported from China in December 2019 and has since spread over the world to become a global pandemic, infecting over 225.7 million people and killing over 4.6 million people till September 16, 2021(2).

To date, there is no highly efficient treatment for COVID-19, making preventive measures such as mask-wearing, hand washing, and social distance the only option to control transmission and the development and deployment of a vaccine is, therefore, one of the most promising strategies in this crisis(3). In early December 2020, the mass vaccination program began and at least 13 different vaccinations across four platforms have been rolled out in countries as of 22 June 2021(4).

The vaccination campaign was launched nationally as well as in all regions in simultaneous high-level events. As per the National Deployment and Vaccination Plan (NDVP) developed following the WHO Prioritizing Roadmap, frontline health workers and support staff, the elderly with underlying conditions and other high-risk groups will be prioritized for vaccination. On 13 March 2021, the Ministry of Health of Ethiopia launched the COVID-19 vaccine (AstraZeneca vaccines produced by Serum Institute of India (SII)) in an event held at Eka Kotebe COVID-19 Hospital where frontline health workers were vaccinated to mark the beginning of the vaccination campaign(5). Ethiopia aims to vaccinate 20% of the population by the end of 2021(5). Vaccine administration started on March 31, 2021, at Adama hospital medical college.

Thus far, all the available data on COVID-19 vaccine side effects has been published by manufacturer-funded studies following the drug authorities' guidelines and monitored by third parties (6). The overall safety of COVID-19 Vaccine AstraZeneca [ChAdOx1nCoV-19 Corona Virus Vaccine (Recombinant)] is based on an interim analysis of pooled data from four clinical trials conducted in the United Kingdom, Brazil, and South Africa(7). About 2/3 of vaccinated individuals develop mild side effects. No causally related serious adverse events (SAE) were caused by the study vaccine(7).

Post-vaccination safety profile is still mandatory to be collected in early vaccination of the community. A lack of vaccine side effect profiles in the area may have a negative impact on vaccine uptake(8). Therefore, this study is aimed at estimating the prevalence of COVID-19 vaccine side effects among the early vaccinated healthcare workers in eastern Ethiopia.

## Methods and Subjects

**Study area and period**: This study was conducted from March to June 2021 at Adama hospital medical college which is located in Adama Town, East Shoa Zone, Oromia regional state in Ethiopia. Adama Hospital Medical College (AHMC) is a teaching referral hospital serving more than six million populations in surrounding regions. COVID-19 vaccine (ChAdOx1 nCoV- 19) was administered at Adama hospital medical college community frontline health workers from March 31, 2021, to April 7, 2021. Health care workers who were vaccinated for Covid-19 were included in the study.

**Study methods and statistical analysis**: The study was a hospital-based Cross-sectional survey using a structured self-administered questionnaire and telephone interview. The sample size for conducting this survey was calculated to be 600 with a 4% margin of error to be conservative and a 95% confidence level in a single population proportion formula. We assumed the prevalence of the side effects after vaccination in the population to be 50%.

To fulfill the required sample size as we don't have control over the total no of HCWs to be vaccinated, and to capture data on immediate post-vaccination reactions, we purposively collected all HCWs coming to the vaccination site till sample size is reached.

Within seven days 802 Health care workers (HCWs) were vaccinated and the first 600 HCWs consented and enrolled in a study. Finally, 540 participants who were able to be contacted by telephone for part two questions and having complete data were subjected for analysis.

**Instrument**: The self-administered questionnaire of this study has two parts, part one for demographic data, was taken at first contact with data collectors during vaccination and part two of the questionnaire (for postvaccination symptoms) was given to participants to take home and daily recording to minimize recall. After the 7th -day participants were interviewed via telephone for side effects from the part two questionnaire. Those who have symptoms till the 8th-day were contacted daily by telephone until symptoms disappear.

The side effect symptoms were selected after reviewing the previous studies, WHO, CDC, and manufacturer side effect profile (4, 7, 15). It has also open-ended spaces for reporting other symptoms not included in the options.

Questionnaires were prepared in English and translated back into Afan Oromo (local language) and translated back into English to check its consistency by two physicians independently and corrections made together for better local language symptom representation. The questionnaire was tested on 20 first vaccinated HCWs and minimal amendments were made before actual data collection.

Data was entered and analyzed using SPSS version 20 computer software. Summary results are presented by frequency tables and graphs. Bivariate analysis like binary logistic regression was done to see the existence of an association between dependent and independent variables. Variables that are significant at a P-value of < 0.25% in binary logistic regression are considered for multiple logistic regressions. Finally, all groups of selected explanatory variables will be fitted to a final model, and the p-value less than 0.05 will be used as a cut-off point for the presence of statistical significance. Adjusted Odds ratio will be used as a measure of association at 95% CI.

**Ethics**: The ethical clearance was obtained from Institutional review board (IRB) of Adama Hospital medical college.

**Operational definitions**: In this study, a vaccine side effect is considered when a new symptom local to the injection site or systemic is developed during the early post-vaccination periods.

SEVERE SYPTOM is a post-vaccine symptom that resulted in the use of pain relievers, requires medical intervention, or that prevents daily activity.

## Results

**Demographic characteristics**: Of 1400 eligible health care workers including supportive staff and medical students, 802 of them were vaccinated with first dose of ChAdOx1 nCoV-19 Corona Virus Vaccine (Recombinant) COVISHIELDTM between 31-March to 6-April, 2021. A total of 540 health care workers and supportive staffs who were recipients of the first dose of ChAdOx1 nCoV- 19 enrolled in the study. The mean age of the participant was 34 years old with a standard deviation of 8.9 years. The majority (60%) of them were aged between 26 to 35 years old. Regarding the gender 351 (65%) of them were males (Table 1). The majority of the participants who took vaccine were doctors 204 (37.8%), then nurses and midwives account (25.7%), and supportive staffs (18.7%). About one-third (27.8%) of HCWs had been tested for covid-19 for either screening purposes or after being symptomatic (Table 1). Of the total tested, 20 (13.3%) were positive for coronavirus with half of them being symptomatic.

**Table 1:** Demographic characteristics of participants, Adama Ethiopia, 2021 (N=540)

Variable	Category	No	%
Age	21–30	257	47.6
	31–40	201	37.2
	41–50	50	9.3
	>=50	32	5.6
Sex	Male	351	65
	Female	189	35
Profession	Doctor	204	37.8
	Nurse	116	21.5
	Supportive staffs	101	18.7
	Academic staffs	30	5.6
	Lab Tech	25	4.6
	Pharmacy	25	4.6
	Midwife	23	4.3
	Anesthesia	10	1.9
	Radiology Tech	6	1.1
Previous covid-19 Test Result	Positive	20	3.7
	Negative	130	24.1
	Not tested	390	72.2

**Figure 1:**
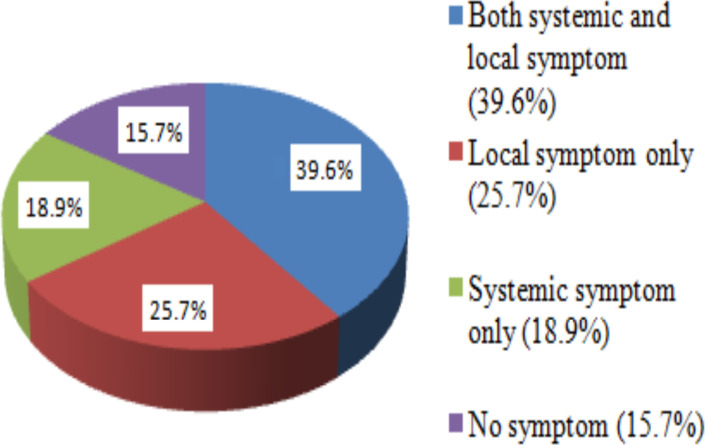
Prevalence of Symptoms reported after taking COVID-19 vaccine, Adama, Ethiopia, 2021

**Prevalence of general side effects after covid-19 vaccination**: The overall any symptom report after the first dose of ChAdOx1 nCoV-19 at Adama was 84.3% and the rest of HCWs reported no symptoms after vaccination. The majority (39.6%) of participants had both systemic and local symptoms and 139 (25.7%) had only local or injection site symptoms and the rest 102 (18.9%) had only systemic symptoms (Figure 1).

The most common symptom reported was injection site pain by 346 (64.1%), followed by fatigue, 193 (35.7%), headache (28.9%), joint pain, 143(26.5%), muscle pain, 116(21.5%), and least reported were skin rash, diarrhea and abdominal pain (Table 2). There were no immediate reactions to the vaccine and the earliest reported time for symptom development was 3 hours. Most adverse effects developed in the first 24 hours and only a few individuals report the adverse effect after the first day.

**Table 2:** Prevalence of reported symptoms after vaccination for SARS COV-2 at Adama, Ethiopia, 2021. (N=540)

Variable		No	%
**Local side effects**	Injection site pain	346	64.1
	Injection site redness	43	8
	Injection site swelling	31	5.7
**Systemic side effects**	Fatigue	193	35.7
	Headache	156	28.9
	Joint pain	143	26.5
	Muscle pain	116	21.5
	Fever	93	17.2
	Chills	47	8.7
	Nausea	36	6.7
	Lack of sleep	36	6.7
	Vomiting	14	2.6
	Oro-pharyngeal pain	13	2.4
	Cough	11	2
	Back pain	7	1.3
	Skin rash	3	0.6
	Diarrhea	3	0.6
	Abdominal pain	3	0.6

**Figure 2 a-j:**
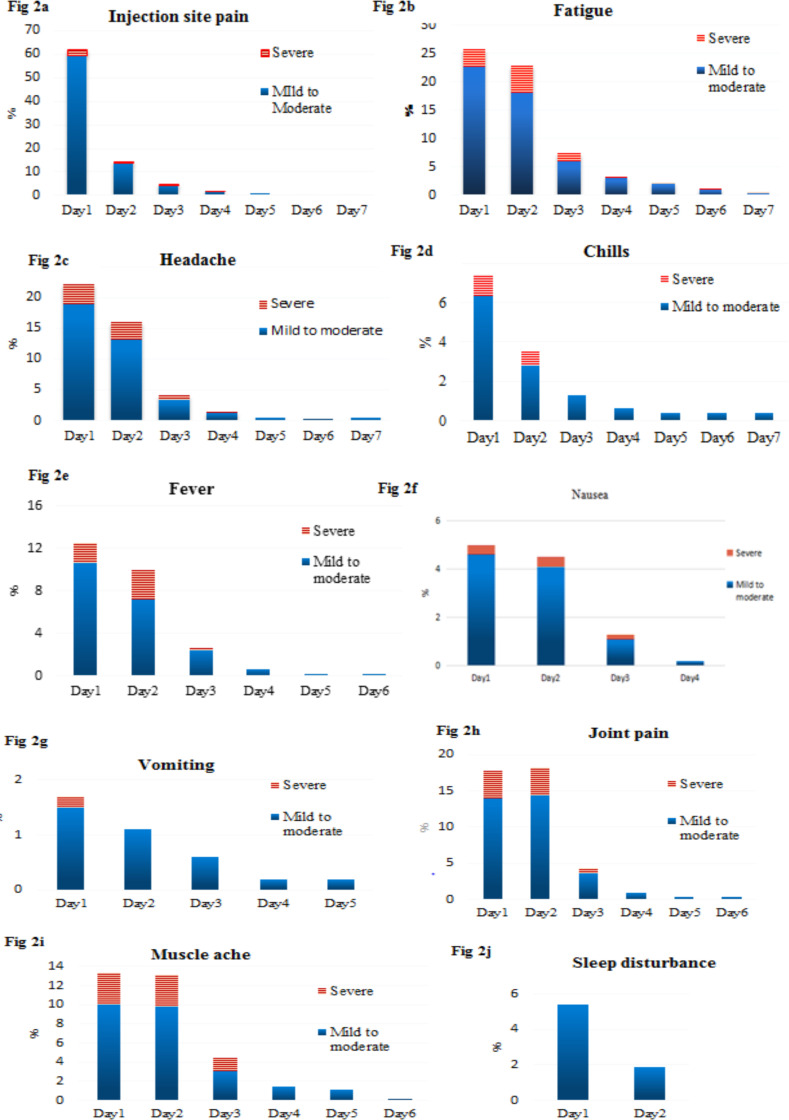
Local and systemic adverse reactions by severity in first 7 days among recipients of the first dose of ChAdOx1 nCoV-19, AHMC, 2021

**Onset and severity of symptoms**: Regarding the onset of symptoms, most participants develop symptoms in the first 24hr after vaccination and few starts to manifest in the second-day post-vaccination. For instance, the injection site pain was reported on the first day by 336 (97.1%), and the rest developed on the second day. Most (93.6%) of the injection site pain was mild to moderate and only 22 (6.4%) of participants reported severe injection site pain requiring rest or anti-pain (Figure 2).

Systemic side effects were categorized as severe if required rest, interruption of daily activity or anti-pain, or other medication utilization. The majority of recipients reported having fatigue on the first day by 122(22.6%) as mild to moderate and 18(3.3%) to be severe. The prevalence of fatigue on the second day was reported by 97(18%) and 26(4.8%) as mild to moderate and severe respectively (Figure 2).

Of the total recipients, 22.2% had experienced headache on the first day which is mild to moderate (18.9%) and 3.3% had a severe headache. The prevalence of headache on the second day was 16.1% and of which 13.1% were mild to moderate and 3% were severe. Two recipients of the vaccine reported mild to moderate headache for more than 1 week (Figure 2).

Fever on the first day was reported by 12.4%, of which 10.6% of recipients reported mild to moderate feverish feeling and did not require medication.

**Figure 3 a-h:**
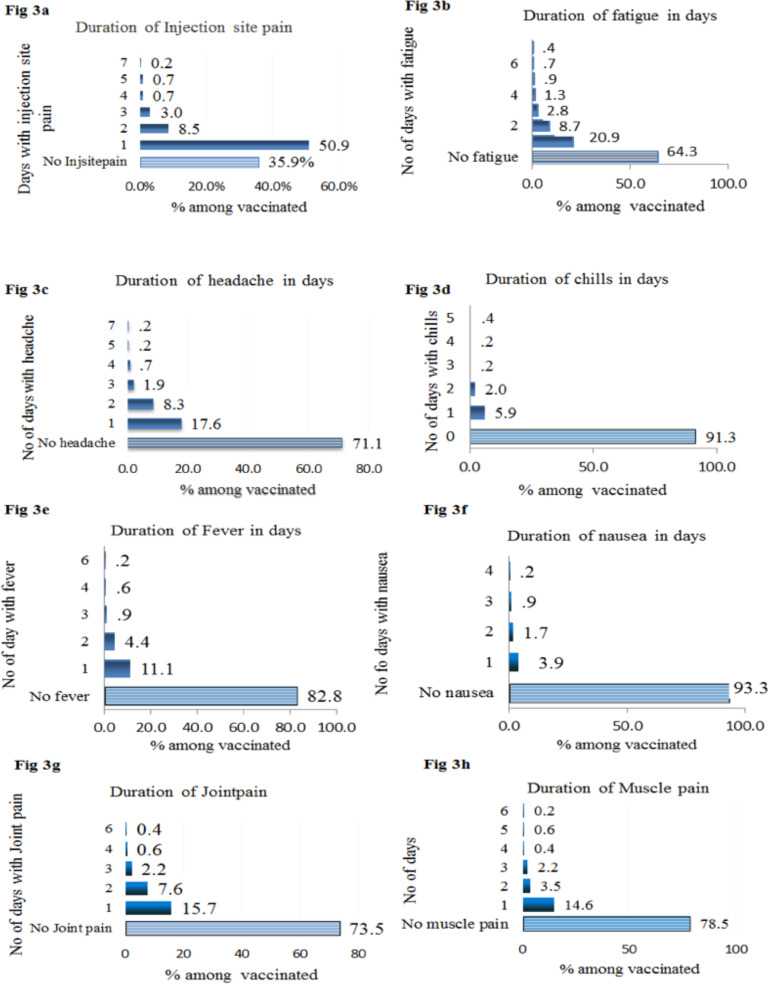
Duration in days of post Covid-19 vaccination side effects, AHMC, 2021.

**Duration of symptoms**: Half (50%) of recipients of the first dose of ChAdOx1 nCoV-19 had injection site pain lasting only on the first-day post-vaccination. 8.5% had injection site pain which lasted for two days, only one of the recipients had local pain which lasted for more than one week. One out of five (20.1%) recipients of the first dose of ChAdOx1 nCoV-19 had fatigue for one day, 47 (8.7%) for two days, 15(2.8%) for three days and only two recipients had developed fatigue lasting one week. About one out of six (17.6%) recipients had headache only for one day. 45(8.3%) had headache that lasted for two days, 10(1.9%) for three days, and only one recipient had headache which lasted for one week. The duration of chills reported was for one day 32(5.9%), for two days 11(2%), and one recipient for more than three days.Fever was reported by 93(17.2%) of total recipients of the first dose of ChAdOx1 nCoV-19. Of these 60(11.1%) of them had a fever for one day, 24(4.4%) had fever lasting for two days and one recipient had a mild fever for six consecutive days. Eighty-five (15.7%) had Joint pain for one day, 41(7.6%) for two days, and 12(2.2%) for three days post-vaccination. One out of seven recipients of ChAdOx1 nCoV-19, 79 (14.6%) had muscle pain of one-day duration, 19(3.5%) two days duration, 12(2.2%) for three days duration (Figure 3).

The general prevalence of symptoms among the age group is not different (p-value= 0.448), similarly, the prevalence of injection site pain which is the most common side effect reported was also similar among age groups (Table 3). However, the prevalence of some systemic symptoms such as muscle ache, fatigue, fever, and headache decrease as age increases (Table 3).

**Table 3:** Vaccine side effects by age group, AHMC, 2021

Variable	Category	Age group				P-value
			
		21–30	31–40	41–50	>=50	
Symptom following vaccination	Yes	221(86%)	166(82.6%)	41(82%)	27(84.4%)	0.448
	No	36(14%)	35(17.4%)	9(18%)	5(15.6%)	
Injection site pain	Yes	169(65.8%)	128(63.7%)	28(56%)	21(65.6%)	0.441
	No	88(34.2%)	73(36.3%)	22(44%)	11(34.4%)	
Fatigue	Yes	94(36.6%)	79(39.3%)	14(28%)	6(18.8)	0.050
	No	163(63.4%)	122(60.7%)	36(72%)	26(81.2%)	
Headache	Yes	83(32.3%)	58(28.9%)	12(24%)	3(9.4%)	0.009
	No	174(67.7%)	143(71.1%)	38(76%)	29(90.6%)	
Fever	Yes	50(19.5%)	35(17.4%)	7(14%)	1(3.1%)	0.030
	No	207(80.5%)	166(82.6%)	43(86%)	31(96.9%)	
Joint pain	Yes	78(30.4%)	47(23.4%)	12(24%)	6(18.8%)	0.061
	No	179(69.6%)	154(76.6%)	38(76%)	26(81.2%)	
Muscle pain	Yes	61(23.7%)	45(22.4%)	8(16%)	2(6.2%)	0.026
	No	196(76.3%)	156(77.6%)	42(84%)	30(93.8%)	

**Figure 4:**
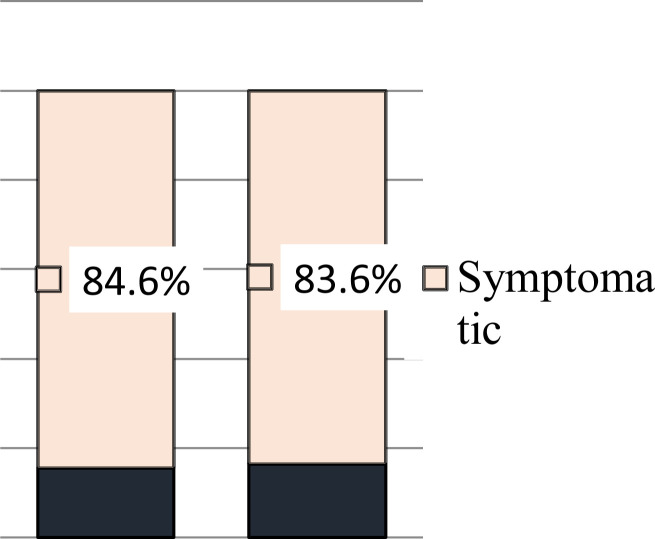
Post-vaccination symptoms by gender, AHMC, 2021.

The existence of an association between gender and symptom reported was assessed using a Chi-square test. There was no significant relationship between vaccine side effects and gender (Figure 4). One hundred and fifty (27.8%) of the vaccine recipient had previous covid-19 testing and only 20 (3.7%) of them had a positive result. Sixteen (80%) of previously Covid-19 infected individuals showed post-vaccine symptoms. The majority 8(40%) showed both systemic and local symptoms and 25% had only local symptoms.

**Unusual reports which need further evaluation includes**: A 45 years old woman reported having severe manifestations of the commonly reported symptoms in others for more than two weeks. A breastfeeding mother reported severe pneumonia-like illness on her child after she took the vaccine while breastfeeding. She assumes the condition of her child was due to vaccination.

There is also one report of increased appetite despite no other symptoms seen in a 43yrs old medical doctor.

## Discussion

Our data suggest that the initial dose of ChAdOx1 nCoV-19 vaccination was safe and well-tolerated by health care workers of Adama Hospital Medical College. There were no major adverse events to ChAdOx1 nCoV-19. The majority of reported adverse effects were mild to moderate in intensity, and they all resolved on their own within the first seven days. There were no statistically significant variations in side effects between genders or age groups. This is in line with the findings of phase 1 and phase 2/3 trials of ChAdOx1 nCoV-19 vaccinations, in which the majority of recipients had minor side effects (7, 9, 10).

The most prevalent side effects described in this study were fatigue, headache, joint and muscular pains, which coincided with phase 1 and 2 clinical trial data from five sites in the UK(9). However, the percentage of occurrences of those symptoms was lower in our study. A similar study conducted in Ethiopia with an online survey yielded similar results, except for a report of 6% severe adverse events among healthcare personnel, which did not appear in our study(11). Comparable studies from India found that after vaccination with the ChAdOx1 nCoV-19 vaccine, there were no major adverse effects among health care professionals(12,13), which is similar to our findings. The rates of side effects, on the other hand, were lower than ours(12,13).

Following vaccination with ChAdOx1 nCov-19, there was a rare development of immunological thrombotic thrombocytopenia and venous thrombosis, according to certain reports(14–17). For instance, five health care professionals who developed venous thrombosis and thrombocytopenia 7 to 10 days after receiving the first dose of the ChAdOx1 nCoV-19 adenoviral vector vaccine against Covid-19 reported with case report data (15). Following such revelations, European governments temporarily halted the use of the Oxford-AstraZeneca vaccine(18). However, the UK Medicines and Healthcare Products Regulatory Agency and the European Medicines Agency conducted extensive reviews, with both agencies confirming that the risk of venous thromboembolism associated with vaccines was no higher than the background risk in the general population, and emphasizing the vaccines' overwhelmingly favorable risk-benefit ratio(19–20). In our cases, none of the healthcare workers had such manifestations.

One of the survey's findings is that only 57% of qualified healthcare providers are immunized in the first phase of vaccination. Several studies in Ethiopia found considerable COVID-19 vaccination hesitancy among healthcare personnel(21, 22). This aversion to the Covid-19 vaccination was also observed in other investigations conducted in different countries (23–26). In Ethiopia, vaccination apprehension exists among the general populace, which could hamper the government's efforts to contain the pandemic and its repercussions(27–30). To address this issue and increase vaccination rates, several strategies have been proposed, including using evidence-based levers and argumentation tools with experts, using behavioral insights to make vaccination more accessible, and finally, assisting early adopters in communicating about their decision to be vaccinated in order to hasten the emergence of pro-vaccination norms (31).

This is one of the first reports on vaccination side effects in immunized Ethiopians. The report will have an impact on understanding the side-effect profiles peculiar to our country, and it will play a constructive role in overcoming the observed vaccine hesitancy. However, it would have been preferable if the side effects related to the second dose had been included in the study to have a complete picture of the vaccine's side effects.

In conclusion, vaccine side effects were common and found to be well-tolerated among the recipients of the first dose of ChAdOx1 nCoV-19 at Adama Hospital Medical College healthcare workers. Local and systemic adverse reactions following vaccination were common and mainly mild to moderate, as described by the manufacturer. There were no anaphylactic or severe hypersensitivity events reported in close proximity to the vaccination. More side effect profiles in different population groups should be studied to detect rare adverse reactions.
